# Supplementary material for: Structural and biochemical evidence for the emergence of a calcium-regulated actin cytoskeleton prior to eukaryogenesis
Source: Commun Biol. 2022 Aug 31;5:890. doi: 10.1038/s42003-022-03783-1 (PMC9433394; doi:10.1038/s42003-022-03783-1)
Supplement: Supplementary file 2 — Supplemental Information [file 42003_2022_3783_MOESM2_ESM.pdf]

Supplementary Information for

Structural and biochemical evidence for the emergence of a calcium-regulated actin cytoskeleton prior to eukaryogenesis.

Caner Akıl[a,b,c*], Linh T. Tran[c*], Magali Orhant-Prioux[d], Yohendran Baskaran[a], Yosuke Senju[c], Shuichi Takeda[c], Phatcharin Chotchuang[f], Duangkamon Muengsaen[f], Albert Schulte[f], Edward Manser[a,b], Laurent Blanchoin[d,e] & Robert C. Robinson[a,c,f,*]

Email: br.okayama.u@gmail.com

This PDF file includes:

Supplementary Figures 1 to 11
SI References

Other supplementary materials for this manuscript include the following:

Supplementary Movies 1 to 4

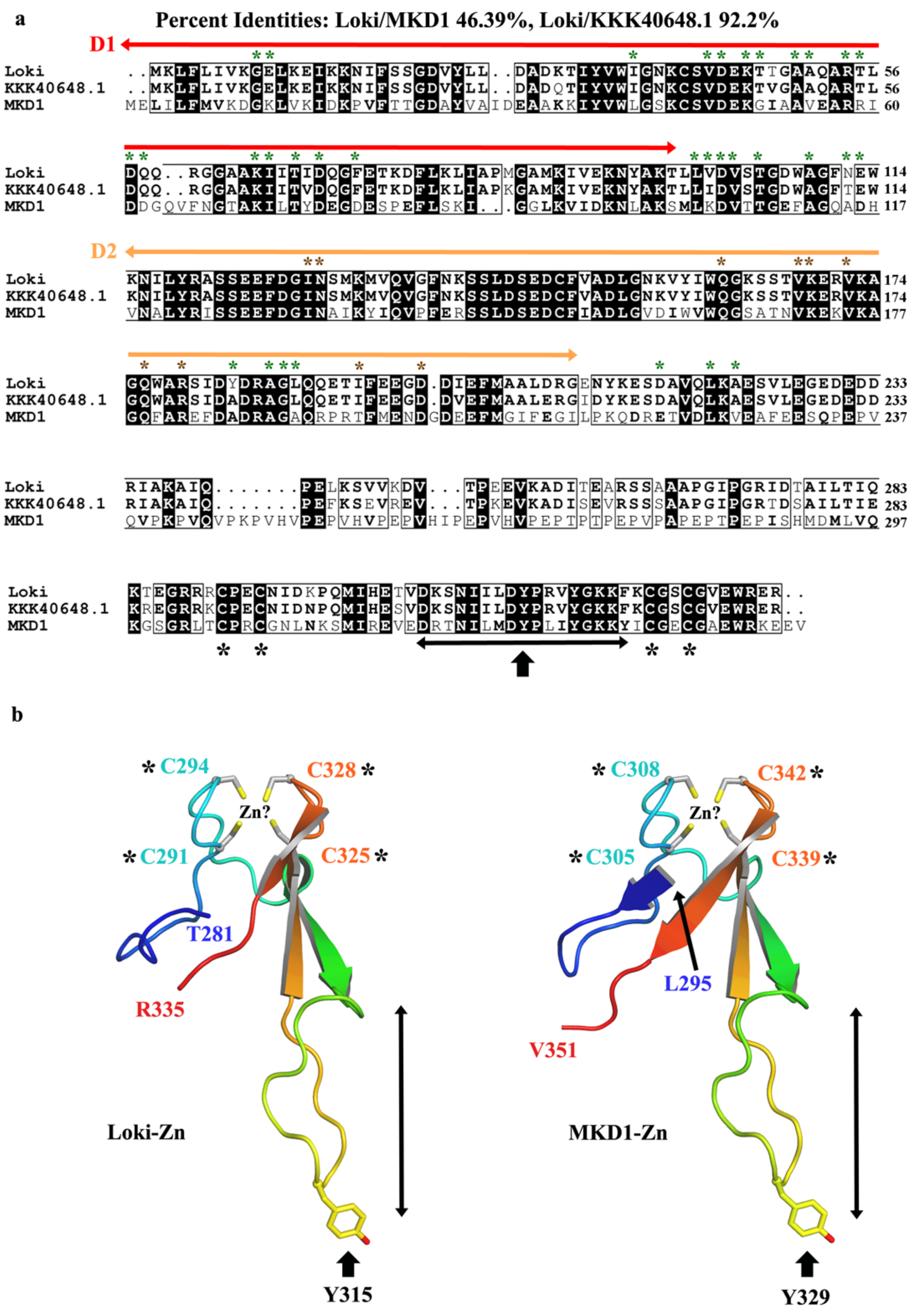
a
Percent Identities: Loki/MKD1 46.39%, Loki/KKK40648.1 92.2%
D1
D2
Loki
KKK40648.1
MKD1
b
C294
C328
Zn?
C291
C325
T281
R335
Loki-Zn
Y315
C308
C342
C305
C339
L295
V351
MKD1-Zn
Y329

c

```
Rabbit MCDE---DETTALVCDNGSGLVKAGFAGDDAPRAVFPSIVGRPRHQGVMVGMG--QKDSY 55
Loki   MAEEESFLNAKALVVDNGTGISKNGFAGEDQPRSVFPTLIGYPKYESIMTDVEHYTREYY 60
MKD1   MVEEYDFLAAKPLVVDNGTGISKNGFAGEDQPRSVFPTLIGYPKYTSIMTDVEHYTREYY 60
       * :*     :. ** ***:*: * ****:* **:***:::* *:: .:*..:    :: *

Rabbit VGDEAQSKRGILTLKYPIEHGIITNWDDMEKIWHHTFYNELRVAPEEHPTLLTEAPLNPK 115
Loki   IGEEAMQLKGVLKLMFPVEHGIIEDWTAMEKIWHYTFYTDLRIDPSEHPVLLTEAPLNPR 120
MKD1   IGEEALQLKGVLKLMFPVEHGVIEDWMAMEKIWHYTFYTDLRVDPSEHPVLLTEAPLNPR 120
       :*:** .  :*:*.* :*:***:* :*  ******:***.:**: *.***.*********:

Rabbit ANREKMTQIMFETFNVPAMYVAIQAVLSLYASGRTTGIVLDSGDGVTHNVPIYEGYALPH 175
Loki   PNREKMAEIMFETFNTPALYVAMQAVLSLYASGRTTGCVIDIGDGVSHVVPIFEGFALSH 180
MKD1   PNREKMAEIMFETFNVPALYVAMQAVLSLYASGRTTGCVIDIGDGVSHVVPIYEGFALSH 180
        *****::*******.**:***:************** *:* ****:* ***:**:** *

Rabbit AIMRLDLAGRDLTDYLMKILTERGYSFVTTAEREIVRDIKEKLCYVALDFENEMATAASS 235
Loki   AIQRIDLAGRDITTYLQRLLRQKGYSFTTSAEKEIVRDIKEKLCYVAIDPEKEMMLSKKV 240
MKD1   AISRLDLAGRDITTYLQRLLRQKGFSFTTSAEKEIVRDIKEKLCYIALDPEKEIMLSKKV 240
       ** *:******:* ** ::* ::*:**.*:**:************:*:* *:*:   : .

Rabbit SSLEKSYELPDGQVITIGNERFRCPETLFQPSFIGMESAGIHETTYNSIMKCDIDIRKDL 295
Loki   AGMEKSYMLPDGETINVGVERFLAPECFFNPSVIGKELEPLDDVIVGAISECDVDLRRDL 300
MKD1   AGMEKVYMLPDGETITVSVERFLAPECFFNPSVIGKELEPLDDVICSAIKQCDVDLRRDL 300
       :.:** * ****:.*.:. *** .** :*:**.** *    :.:.  .:* :**:*:*:**

Rabbit YANNVMSGGTTMYPGIADRMQKEITALAPSTMKIKIIAPPERKYSVWIGGSILASLSTFQ 355
Loki   YSNIVLSGGSTMFPGIKERLTKEIKEQIPESVDVKIIAPPERMYSVWIGGSILSSLKTFH 360
MKD1   YGNIVLSGGSTMYPGLKERLTKEIKEQIPESVDVKIIAPPERMYSVWIGGSILSSLKTFQ 360
       *.* *:***:**:**: :*: ***.   *.::.:******** **********:**.**:

Rabbit QMWITKQEYDEAGPSIVHRKCF 377
Loki   RMWVTRREYKEMGPQVIHR-CF 381
MKD1   RMWVTRREYKEIGPSIIHR-CF 381
       :**:*::**.* **.::** **
```

Supplementary Figure 1 | Comparison of the Loki and MKD1 2DGel sequences. (**a**) Sequence alignment. Domain 1 (D1, red) and domain 2 (D2, orange) are indicated by arrows. Asterisks above the alignment signify residues that contact the actin subunits in the Loki2DGel complex, lower actin (green) and upper actin (brown). Insertions and substitutions between Loki and MKD1 sequences, such as F72D and V99K, likely provide the basis for the differences in activity. The second 2DGel from Loki GC14_75 (KKK40648.1) is included. (**b**) Cartoon representation of the AlphaFold2[1] predicted structures of the potential Zn^{2+}-binding domains from C-termini of Loki2DGel and MKD1-2DGel. The cartoon is shown in rainbow colors blue-to-red along the amino acid chains. The double-headed arrow highlights a conserved region, which is predicted to form a protrusion with a tyrosine at the tip (wide arrow). Asterisks indicate the potential Zn^{2+}-binding residues. These features are indicated are also indicated below the sequence alignment in (a). (**c**) Sequence alignment of the Loki (KKK41204.1) and MKD1 (WP_147662055.1) actins with rabbit actin (P68135). The rabbit actin sequence is that of the predicted sequence from the gene, rather than the post-translational modified (PTM) sequence. 2 should be subtracted from the residue numbers for the PTM numbering. Asterisks above the alignment signify residues that contact the Loki2DGel in the Loki2DGel/2 actin complex, lower actin (green) and upper actin (brown). These binding-site residues are identical for Loki and MKD1 actins, and highly conserved with rabbit actin. Asterisks, colons and dots below the alignment indicate conservation in amino acids. Loki and MKD1 actins share 91.6% identity with each other, and 59.3-59.8% identity with rabbit actin.

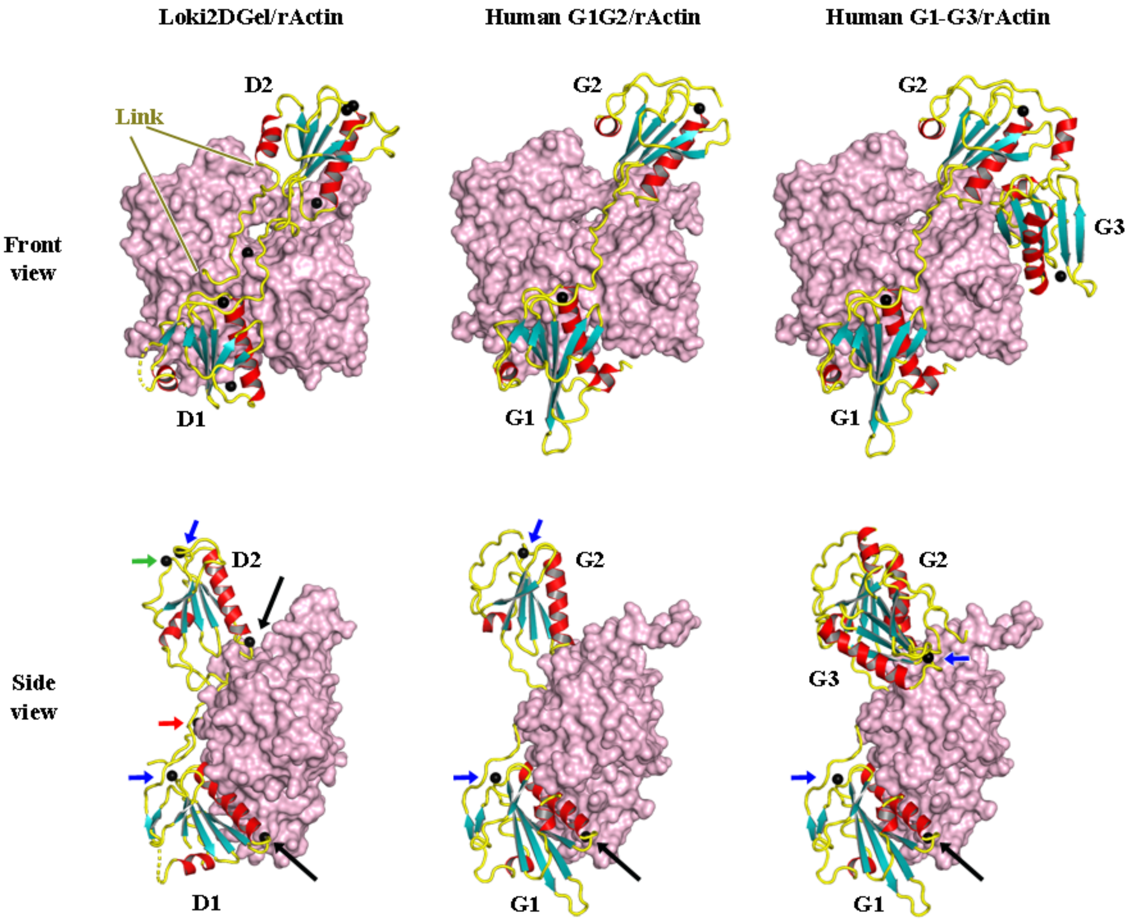

Supplementary Figure 2 | Comparison of the actin and calcium interactions of Loki2DGel with human gelsolin G1-G3[2]. Human G1G2/rActin is the same structure as G1-G3/rActin with gelsolin domain G3 removed for ease of comparison with Loki2DGel. Front view: Highlights the stabilization of D2/G2 in binding actin (pink). Loki2DGel achieves this via the Link region binding to actin and human G1-G3 via G3 binding to actin. Side view: Highlights the calcium-binding sites (arrows). Black, Type I sites; blue, Type II sites; red, LVDV motif site; and green, novel site. The Type II site in G2 in G1-G3 is obscured by G3 in this orientation.

MKD1

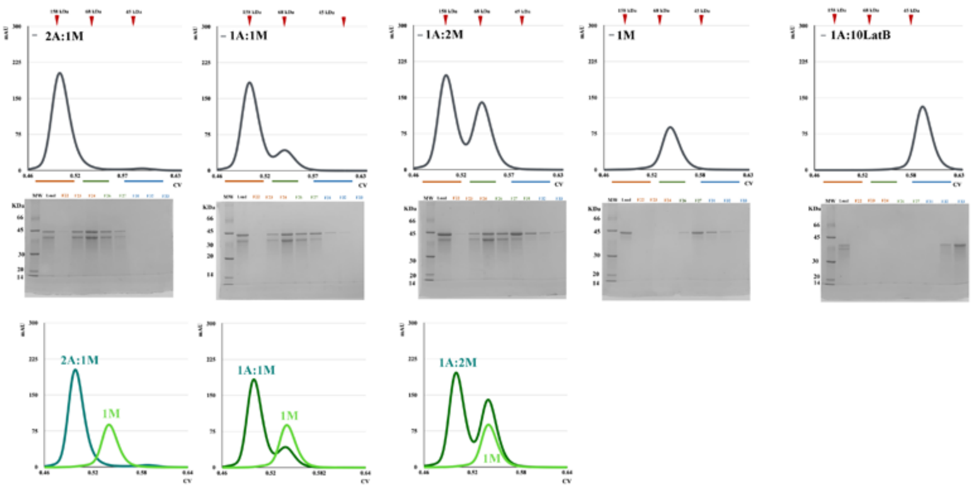

Heimdall

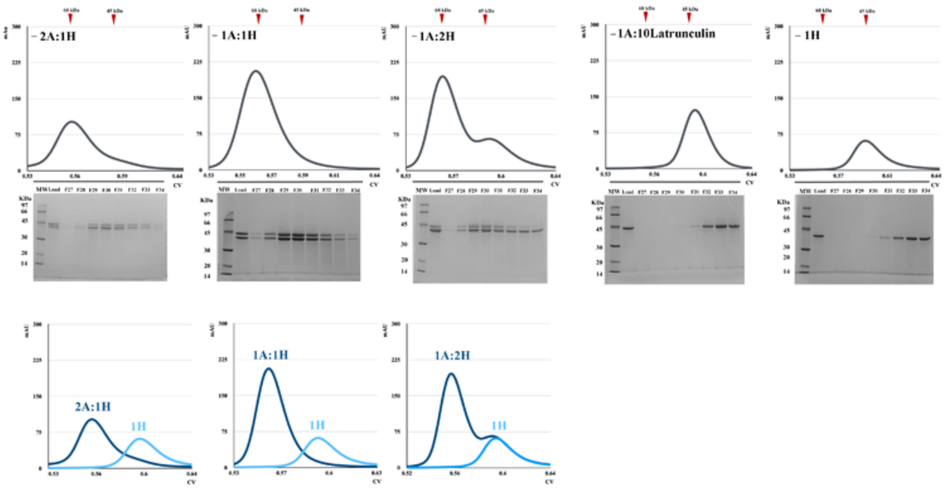

Molecular weight marker

Column volume	KDa	Protein
0.51	158	Aldolase
0.55	68	Albumin from bovine serum
0.58	45	Albumin from hen egg
0.65	25	Chymotripsinogen A

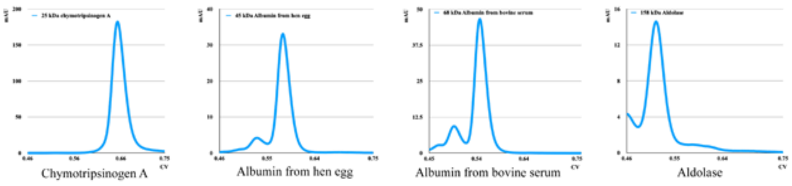

Supplementary Figure 3 | Gel filtration profiles of 2DGels alone or as complexes with actin, and calibration profiles for the MW markers. Supplemental information for Fig. 4a-d.

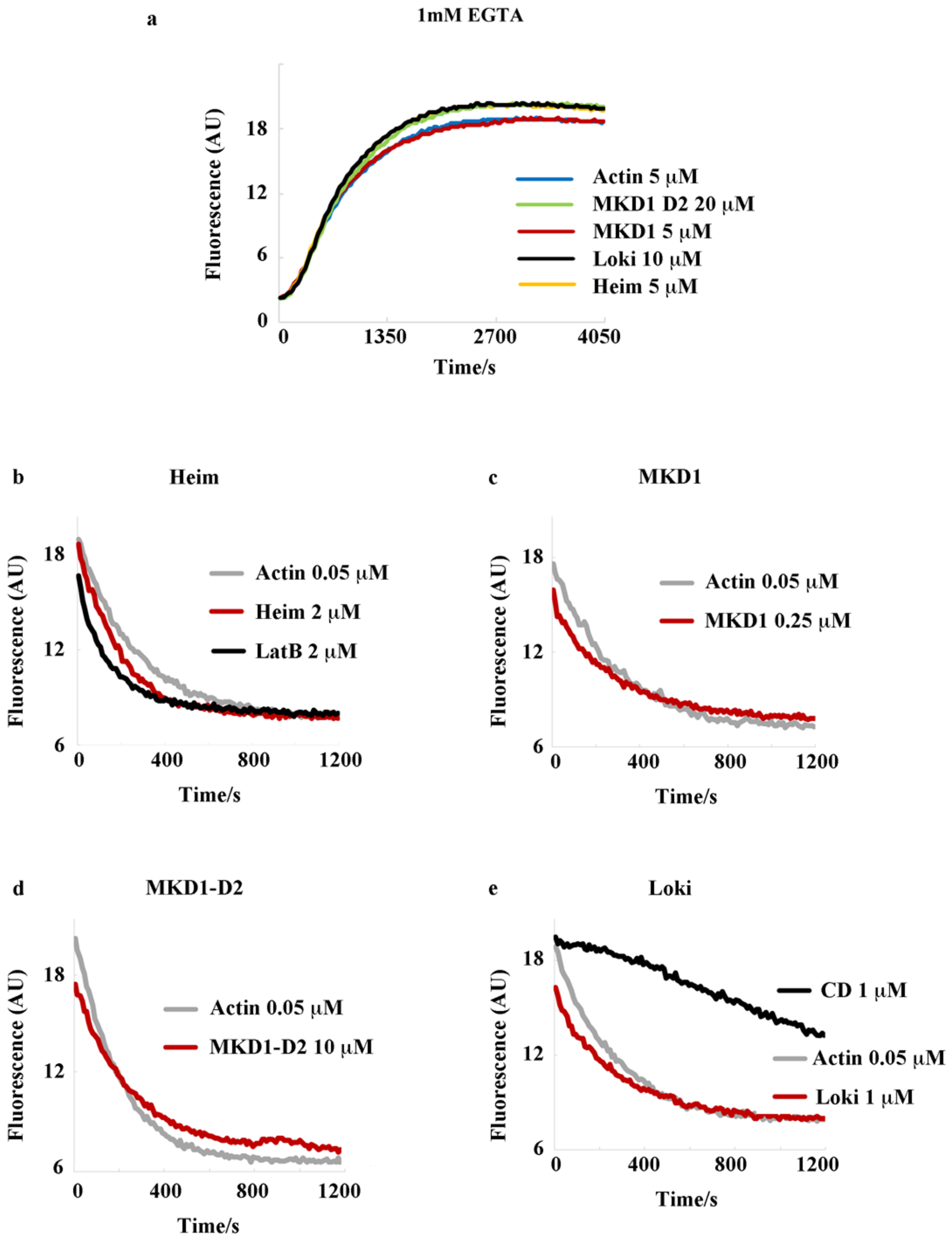

Supplementary Figure 4 | Pyrene actin polymerization and depolymerization assays in the presence of 1 mM EGTA. (**a**) EGTA actin polymerization control curves for Fig. 4e-h. (**b-e**) EGTA actin depolymerization control curves for Fig. 4i-h.

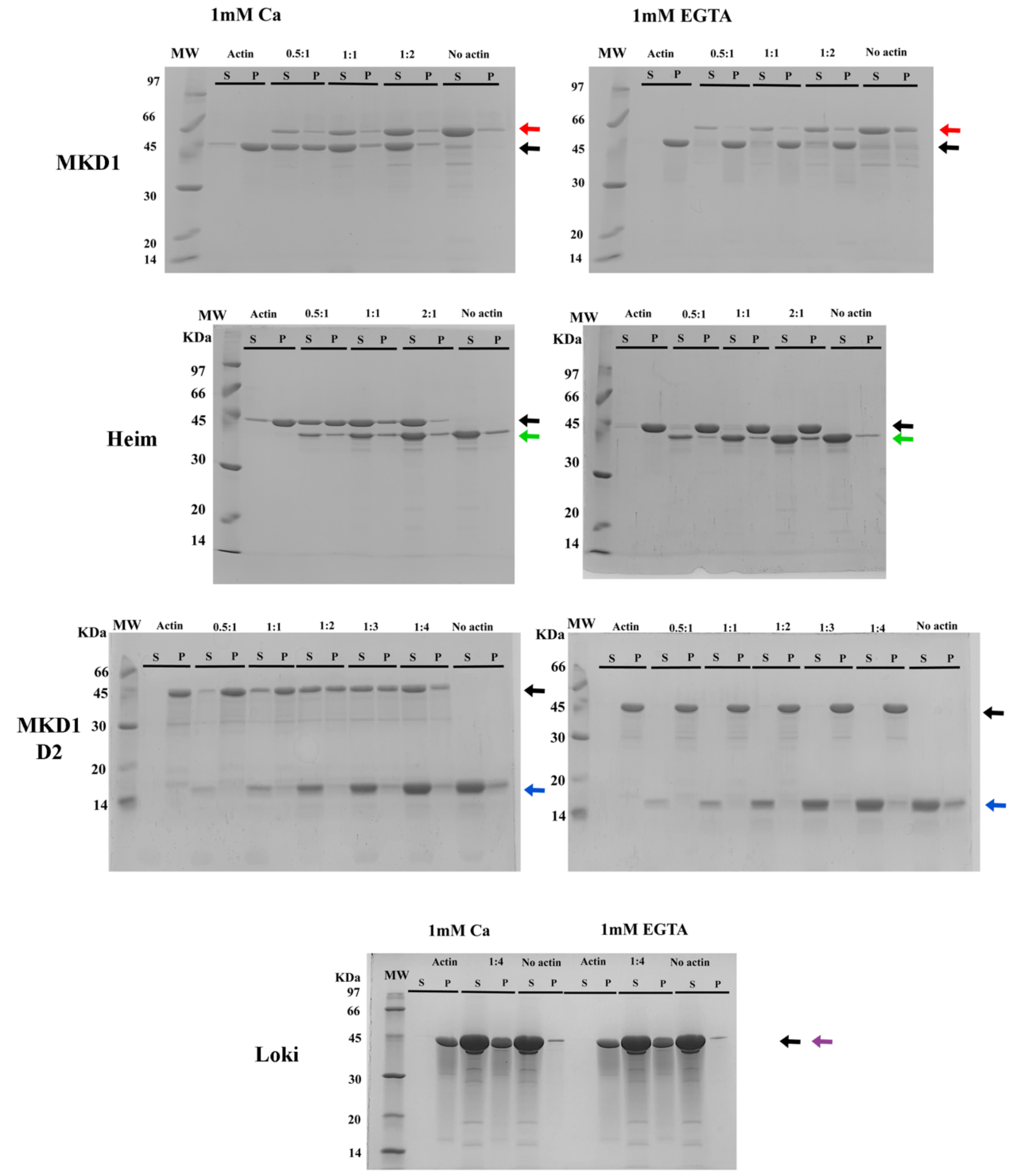

Supplementary Figure 5 | SDS PAGE analysis of high speed sedimentation of F-actin treated with Asgard 2DGels. Accompaniment to Fig. 5a-d. The positions of actin migration are highlighted (black arrows), MKD1-2DGel (MKD1, red arrow), Heim2DGel (Heim, green arrow), MKD1-D2 (blue arrow), and Loki2DGel (Loki, purple arrow).

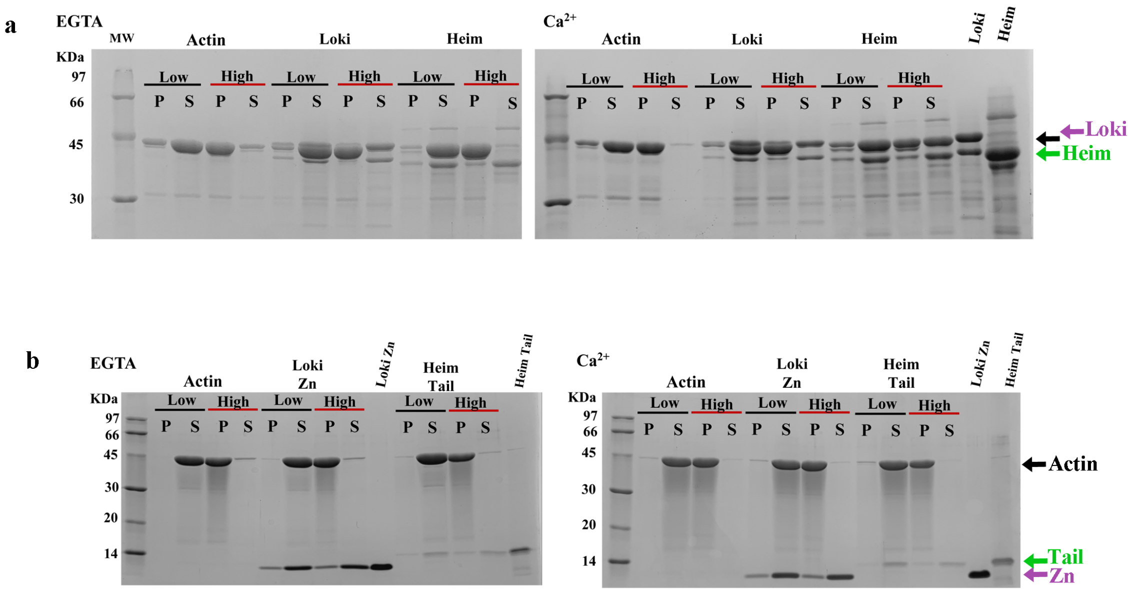

Supplementary Figure 6 | Sedimentation analysis of 2DGels. (**a**) SDS PAGE analysis of high and low speed sedimentation of F-actin treated with Loki and Heim 2DGels (1:1 ratio). Actin sediments at high speed but not at low speed indicating a lack of filament bundling under the conditions tested. (**b**) SDS PAGE analysis of high and low speed sedimentation of Loki-Zn and Heim-Tail with F-actin. The sedimentation patterns of Loki-Zn and Heim-Tail appear similar in the low and high speed centrifugations, indicating a lack of filament binding, since the actin moves from soluble to pellet under these two regimes.

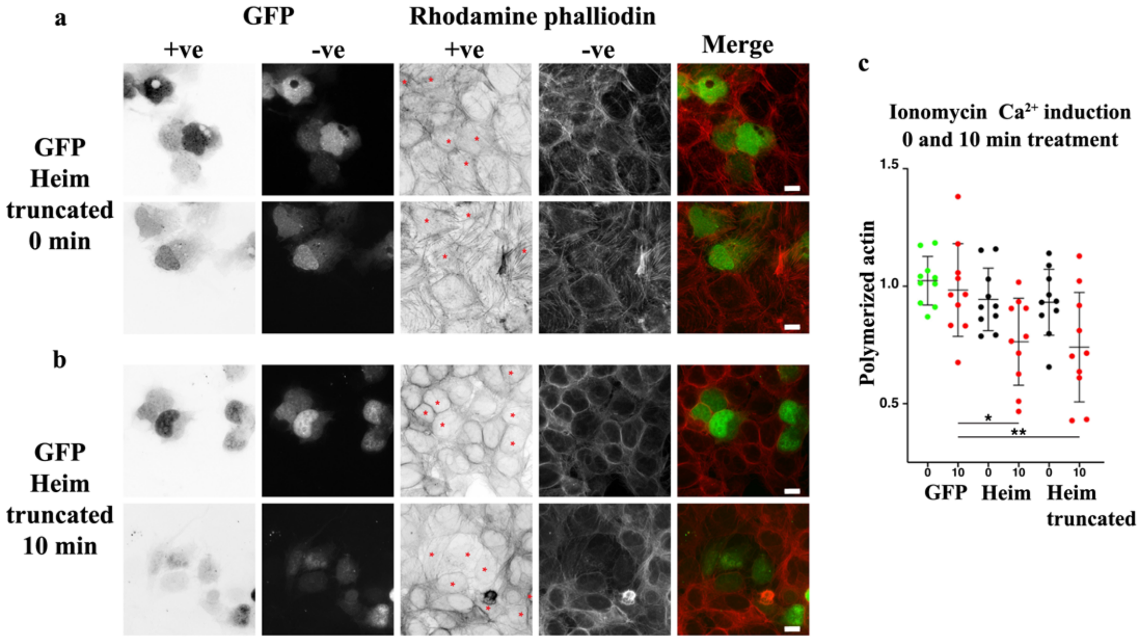

Supplementary Figure 7 | Cellular activity of Heim2DGel without the Tail. (**a**,**b**) U2OS cells expressing a hybrid GFP construct fused to Heim2DGel without the Tail (Heim truncated) imaged before (0 min) or after (10 min) treatment with ionomycin to release cellular calcium stores. (**c**) The relative amounts of F-actin in the GFP, GFP-Heim, and GFP-Heim truncated transfected cells, judged by rhodamine phalloidin staining. Scale bars = 20 μm. Accompaniment to Fig. 6a.

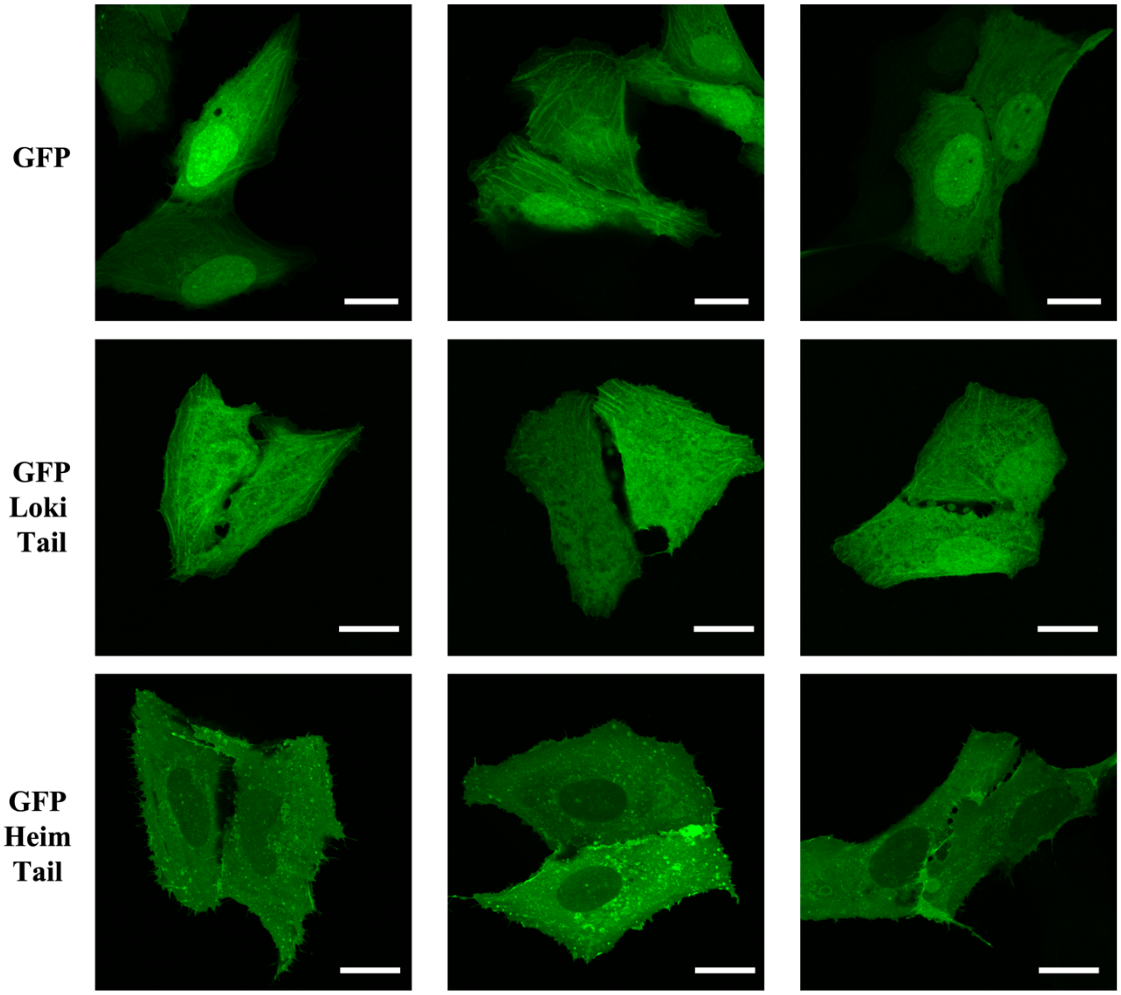

Supplementary Figure 8 | Examples of the cellular localization of the C-terminal Tails. Accompaniment to Fig. 6c. Scale bars = 20 μm.

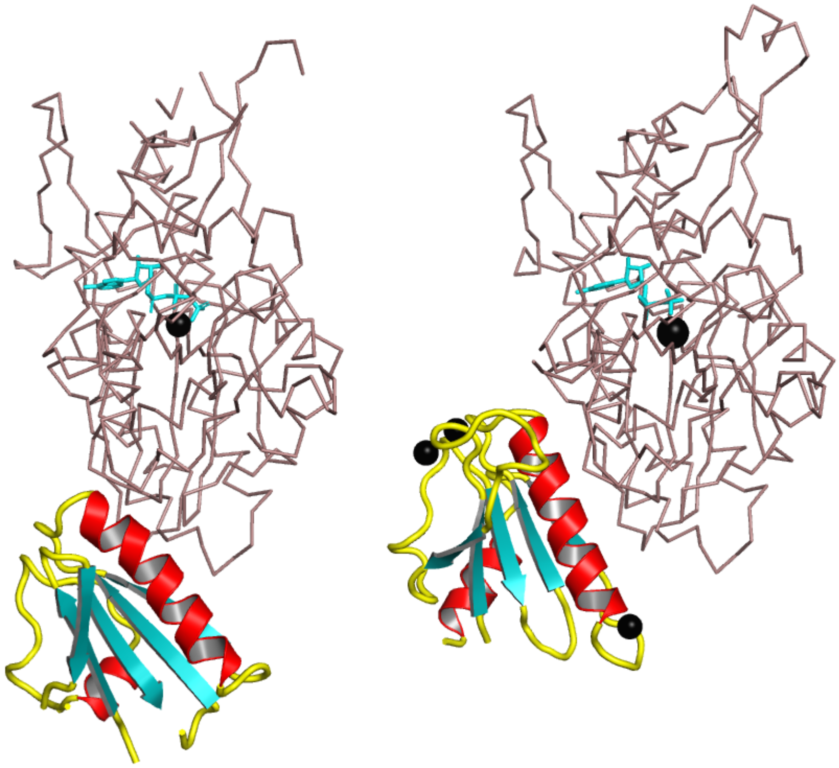

Supplementary Figure 9 | Comparison of the interaction sites of Thor ProGel and Loki2DGel domain 2 (D2) on rabbit actin (brown). The activity of the isolated MKD1-2DGel-D2 is calcium controlled (Fig. 4g and Supplementary Fig. 4a). ProGel represents a model of the ancestral one domain gelsolin protein. Whereas, Loki2DGel-D2 represents a model of the ancestral one domain gelsolin protein on gaining calcium control, prior to the gene duplication to form 2DGel. Examples of a single domain gelsolin with Type I and II calcium binding residues are not present in the current sequence databases, suggesting that this protein has been lost from the Asgard genomes.

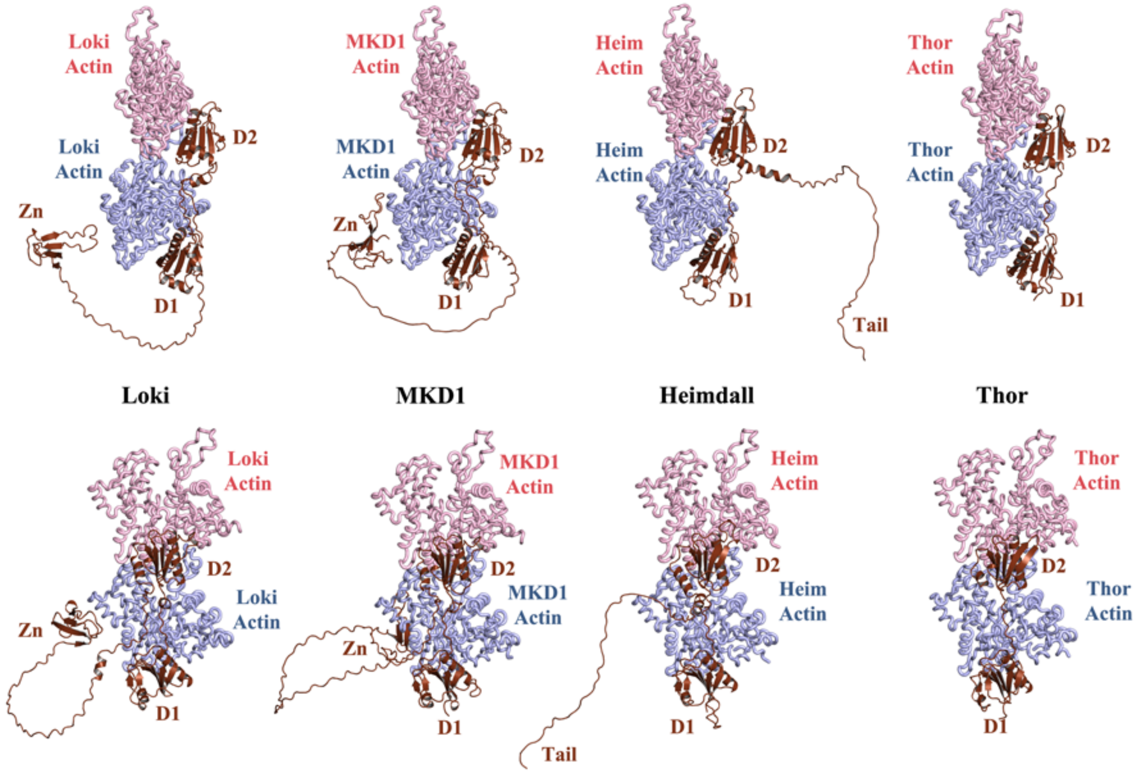

Supplementary Figure 10 | AF2 predictions of the structures of Asgard 2DGels with two Asgard actin subunits. AF2 predicts a common binding site for 2DGel D2 between two actin subunits for the Asgard 2DGels from Fig. 1a. The lower Asgard actins (light blue) are in F-actin conformations, and the upper Asgard actins (light pink) are in G-actin conformations. The Loki and MKD1 Zn domains do not form close contacts with actin, and adopt different orientations, which vary in position in AF2 calculation repetitions. The Loki Link and Heim Tail regions do not have ordered structure predictions in AF2. Heim actin (OLS19029.1 LC_2); Loki actin (KKK41204.1 GC14_75); MKD1 actin (WP_147662055.1) and Thor actin (KXH72853.1 SMTZ1-83). The calcium-binding loop in D2 of Heim2DGel is close to the surface of actin, suggesting that these calcium sites are important for the interaction. The Loki2DGel/LokiActin predicted structure provides some clues to the low activity of Loki2DGel for rActin. Loki2DGel residue E9 is predicted to form a charge:charge interaction with LokiActin K375. rActin has S350 in this position, which does not interact in the crystal structure. LokiActin, and MKD1-Actin, has a two residue insert in the D-loop (H54-Y55, Supplementary Fig. 1c) relative to rActin, which is predicted to form a π:π interaction with Y182 from Loki2DGel. The absence of these interactions may lower the affinity of Loki2DGel for rActin below that needed to compete with rActin subunit interactions, resulting in low activity in the biochemical assays.

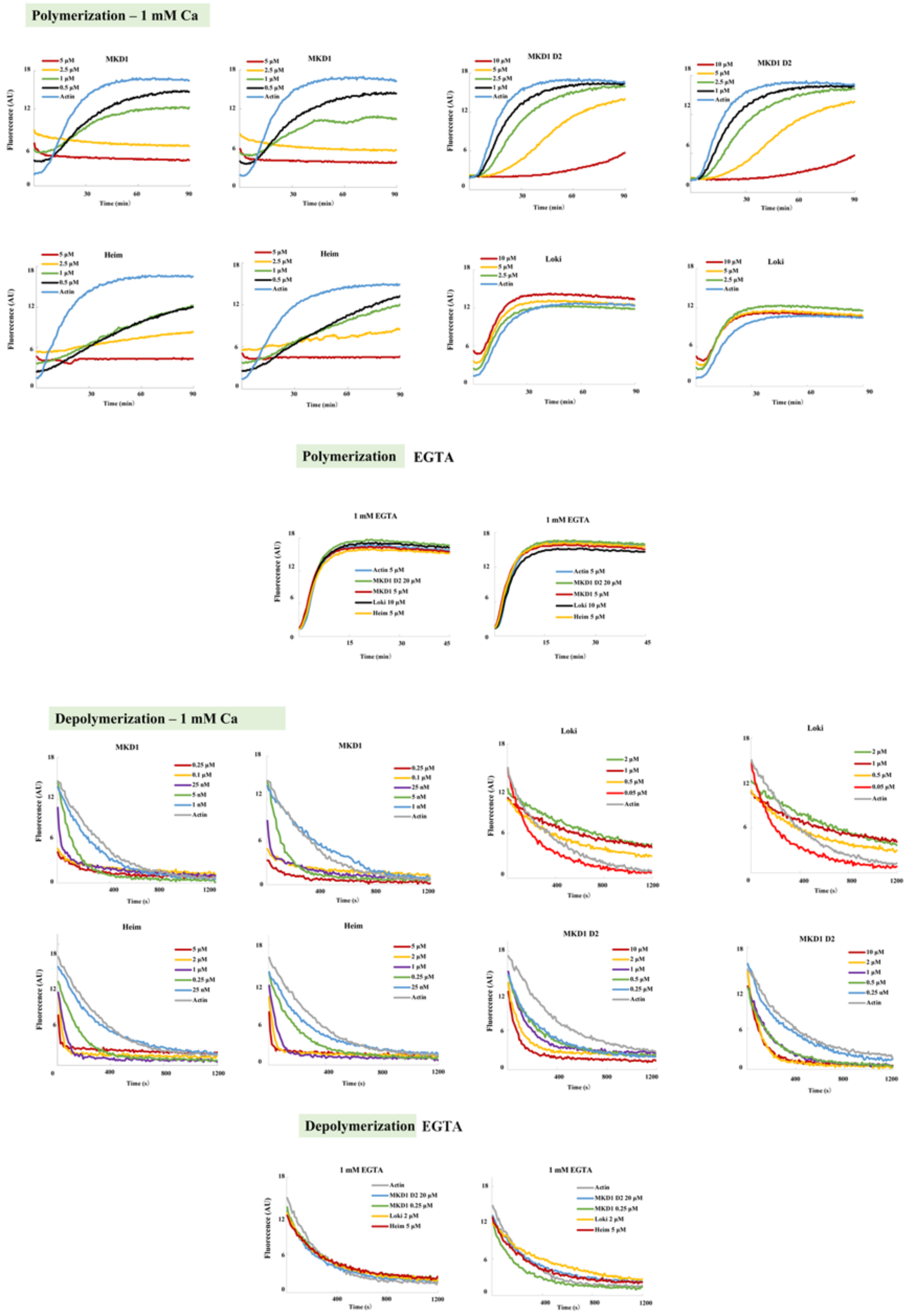

Supplementary Figure 11 | Repetitions of the polymerization and depolymerization assays.

SI References

1. Jumper, J. *et al.* Highly accurate protein structure prediction with AlphaFold. *Nature* **596**, 583–589 (2021).
2. Nag, S. *et al.* Ca2+ binding by domain 2 plays a critical role in the activation and stabilization of gelsolin. *Proc. Natl. Acad. Sci. U. S. A.* **106**, 13713–8 (2009).
