# Supplementary material for: Structural and biochemical evidence for the emergence of a calcium-regulated actin cytoskeleton prior to eukaryogenesis
Source: Commun Biol. 2022 Aug 31;5:890. doi: 10.1038/s42003-022-03783-1 (PMC9433394; doi:10.1038/s42003-022-03783-1)
Supplement: Supplementary file 3 — Description of Additional Supplementary Files [file 42003_2022_3783_MOESM3_ESM.pdf]

Description of Additional Supplementary Files

File name: Supplementary Movie 1
Description: The effect of Loki2DGel on actin filaments.

File name: Supplementary Movie 2
Description: The effect of MKD1-2DGel on actin filaments.

File name: Supplementary Movie 3
Description: The effect of MKD1-2DGel on actin filaments. Shortened and slowed down version of Movie 2.

File name: Supplementary Movie 4
Description: The effect of Heim2DGel on actin filaments.
